# DOES THE NUMBER OF DISEASES OR DISEASE SEVERITY AFFECT THE AGING ATTITUDES IN HEALTH RETIREMENT STUDY?

**DOI:** 10.1093/geroni/igac059.1977

**Published:** 2022-12-20

**Authors:** Jung Da Chang

**Affiliations:** Miami University, Oxford, Ohio, United States

## Abstract

The number of older adults is increasing worldwide, coupled with an increase in chronic disease. Evidence has shown a relationship between health conditions and changes in attitudes toward own aging (ATOA) and thus may contribute to a negative ATOA among older adults with more health conditions. Additionally, studies also show that ATOA can affect the health of older people and become a vicious circle. To promote a healthier life for older adults, it is important to assist them to maintain a more positive ATOA. Therefore, the purpose of this study wants to determine the relationship between the diseases number and ATOA for older adults (N = 6303). Although the relationship between the number of the diseases has been discussed in previous studies, this study will use 2012 and 2016 waves Health and Retirement Study data to update the relationship by using generalized estimating equation. To understand whether it is the disease severity that affects ATOA, this study uses Charlson Comorbidities Index to measure the burden of diseases and use structural equation models to understand the relationship between variables and do the model pathway analysis. The initial results show that an individual with a greater number of diseases (p < .01, r=-.039), and a higher score of CCI have more negative ATOA (p < .01, r=-.04). These findings suggest both the number and severity of diseases contribute to ATOA in older adults. Furthermore, the CCI provides a method to identify the disease severity and can be used in the national databases.

